# Supplementary figures and images for: Horizontal and vertical movements of starry smooth-hound Mustelus asterias in the northeast Atlantic
Source: PLoS One. 2020 Oct 28;15(10):e0239480. doi: 10.1371/journal.pone.0239480 (PMC7592766; doi:10.1371/journal.pone.0239480)

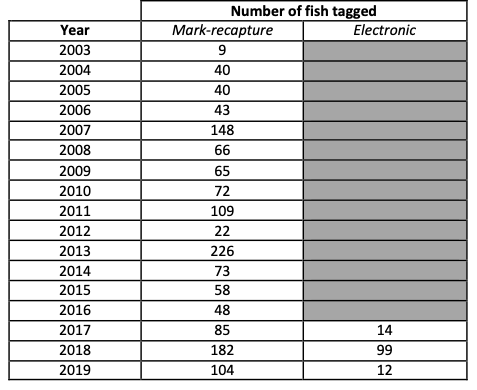

Supplement: S1 Table — Number of M. asterias tagged and released with mark-recapture and electronic tags per year. (PNG) [file pone.0239480.s001.png]

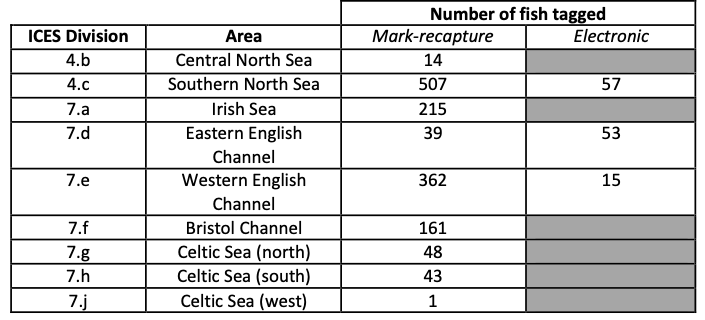

Supplement: S2 Table — Number of M. asterias tagged and released with mark-recapture and electronic tags by ICES Division. (PNG) [file pone.0239480.s002.png]

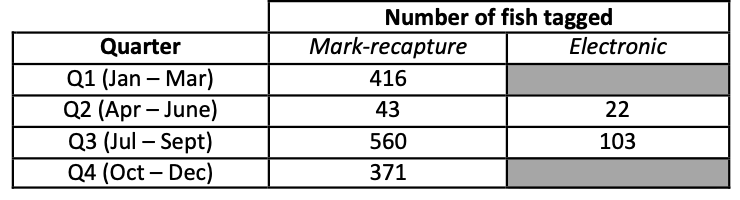

Supplement: S3 Table — Number of M. asterias tagged and released with mark-recapture and electronic tags per quarter. (PNG) [file pone.0239480.s003.png]

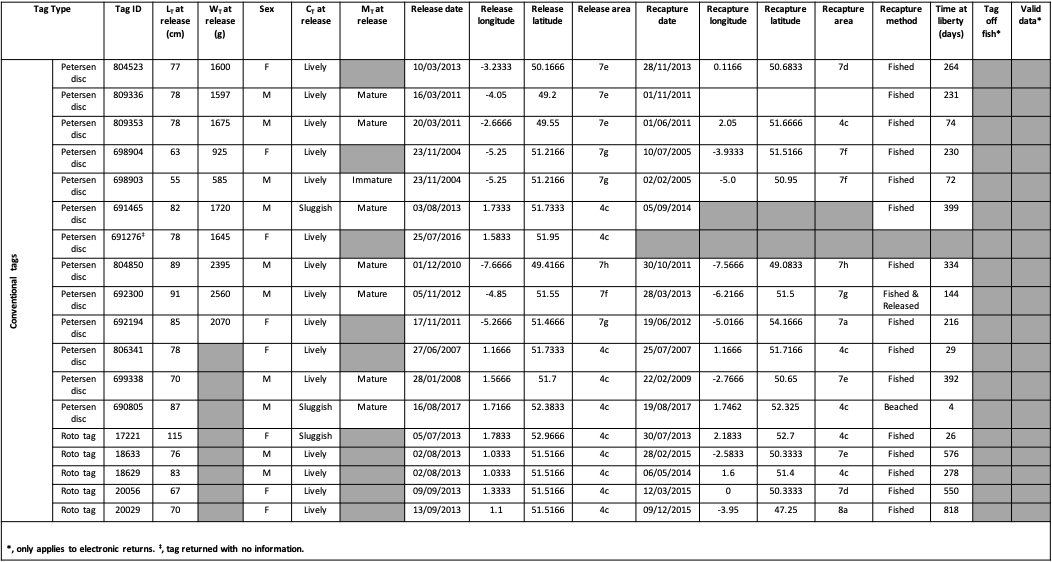

Supplement: S4 Table — Release and recapture information for the 18 returned mark-recapture tags. (PNG) [file pone.0239480.s004.png]

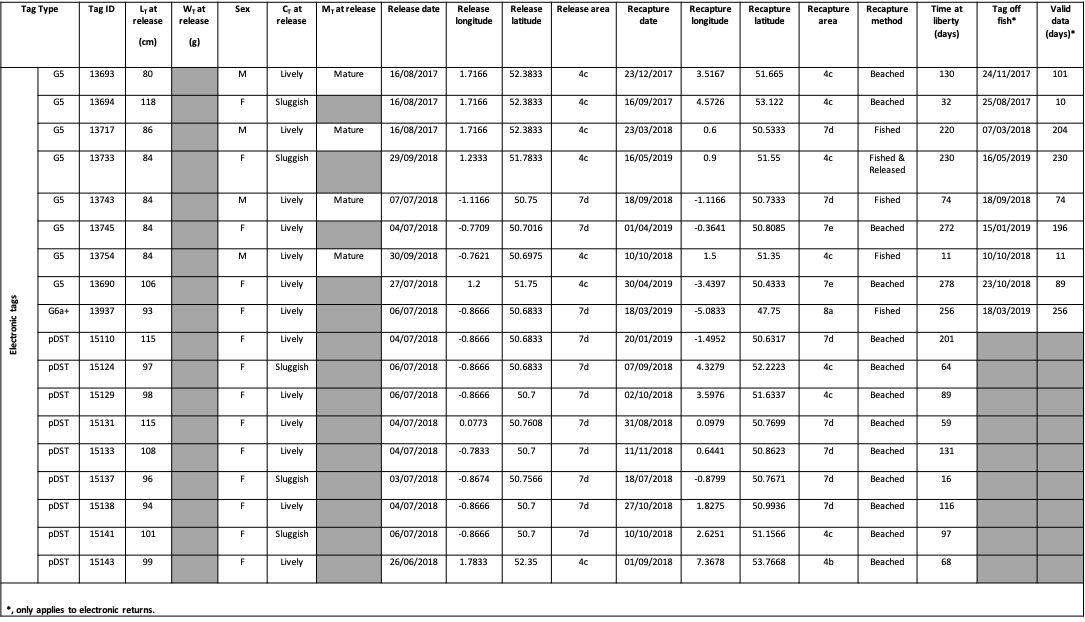

Supplement: S5 Table — Release and recapture information for the 18 returned electronic tags. (PNG) [file pone.0239480.s005.png]

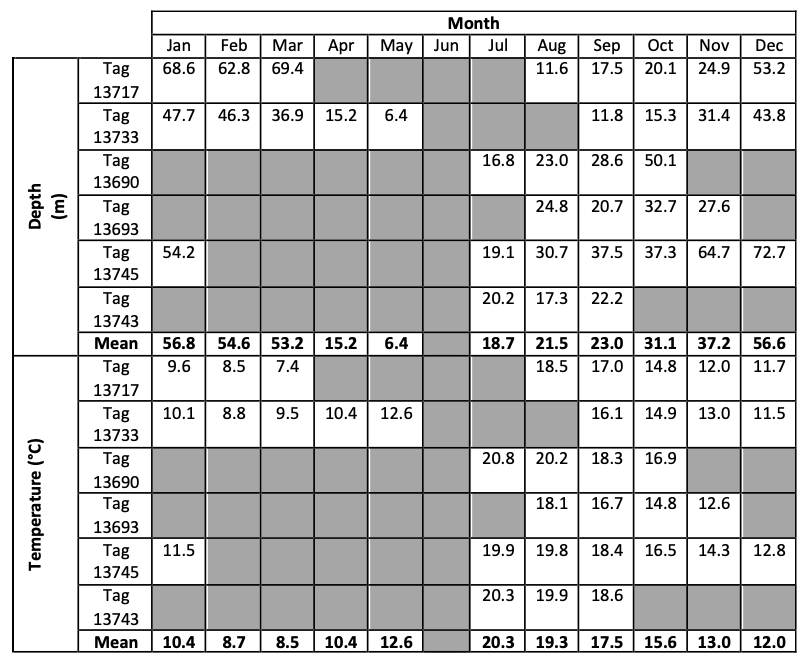

Supplement: S6 Table — Average depth (m) and temperature (°C) measurements recorded per month from M. asterias tagged with electronic tags. Values are presented per individual and as averages across the six individuals. (PNG) [file pone.0239480.s006.png]

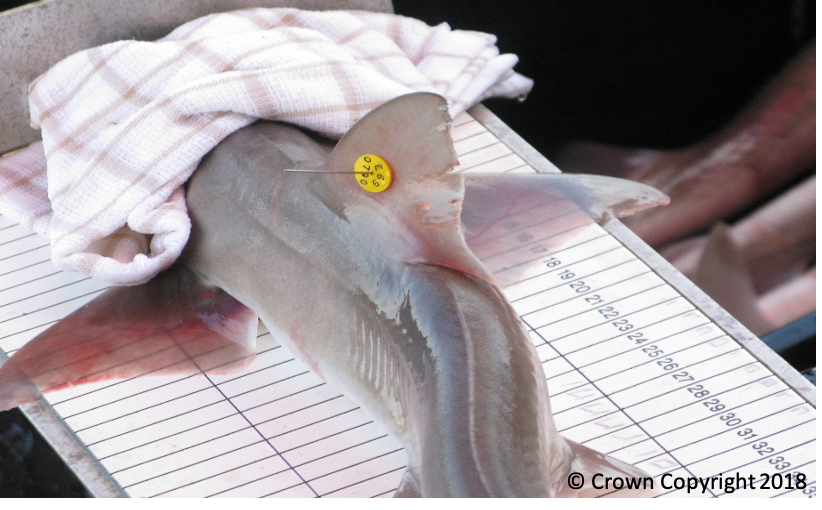

Supplement: S1 Fig — M. asterias in the process of being tagged with a mark-recapture Petersen disc. Shown is the yellow disc listing the tags unique identification number. (PNG) [file pone.0239480.s007.png]

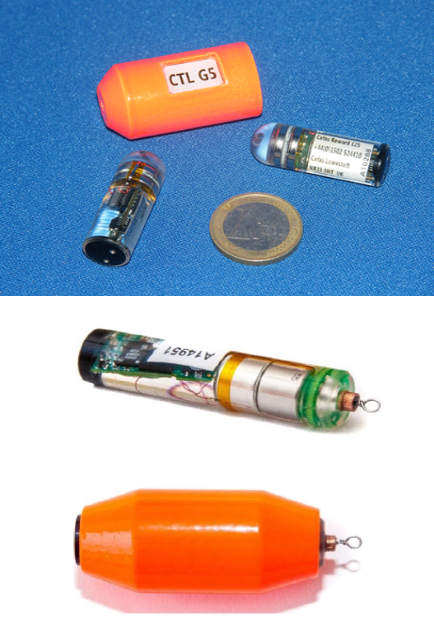

Supplement: S2 Fig — G5 (top) and pDST (bottom) electronic tags. Both tags were fitted with a float jacket (external orange layer) to maximise return rates. Photos taken from https://www.cefastechnology.co.uk/. (PNG) [file pone.0239480.s008.png]

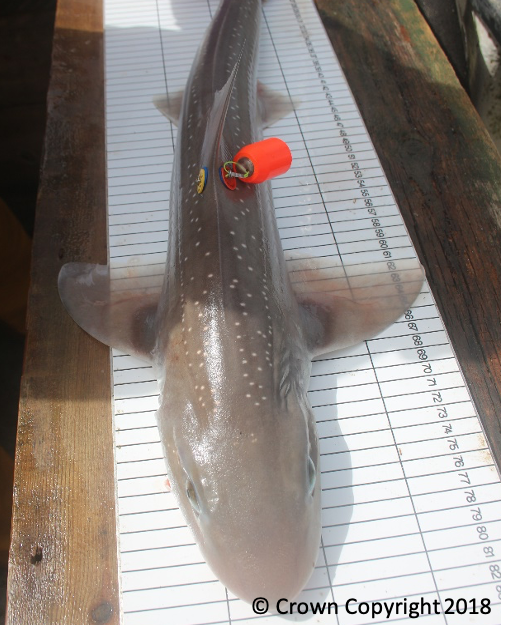

Supplement: S3 Fig — (PNG) [file pone.0239480.s009.png]

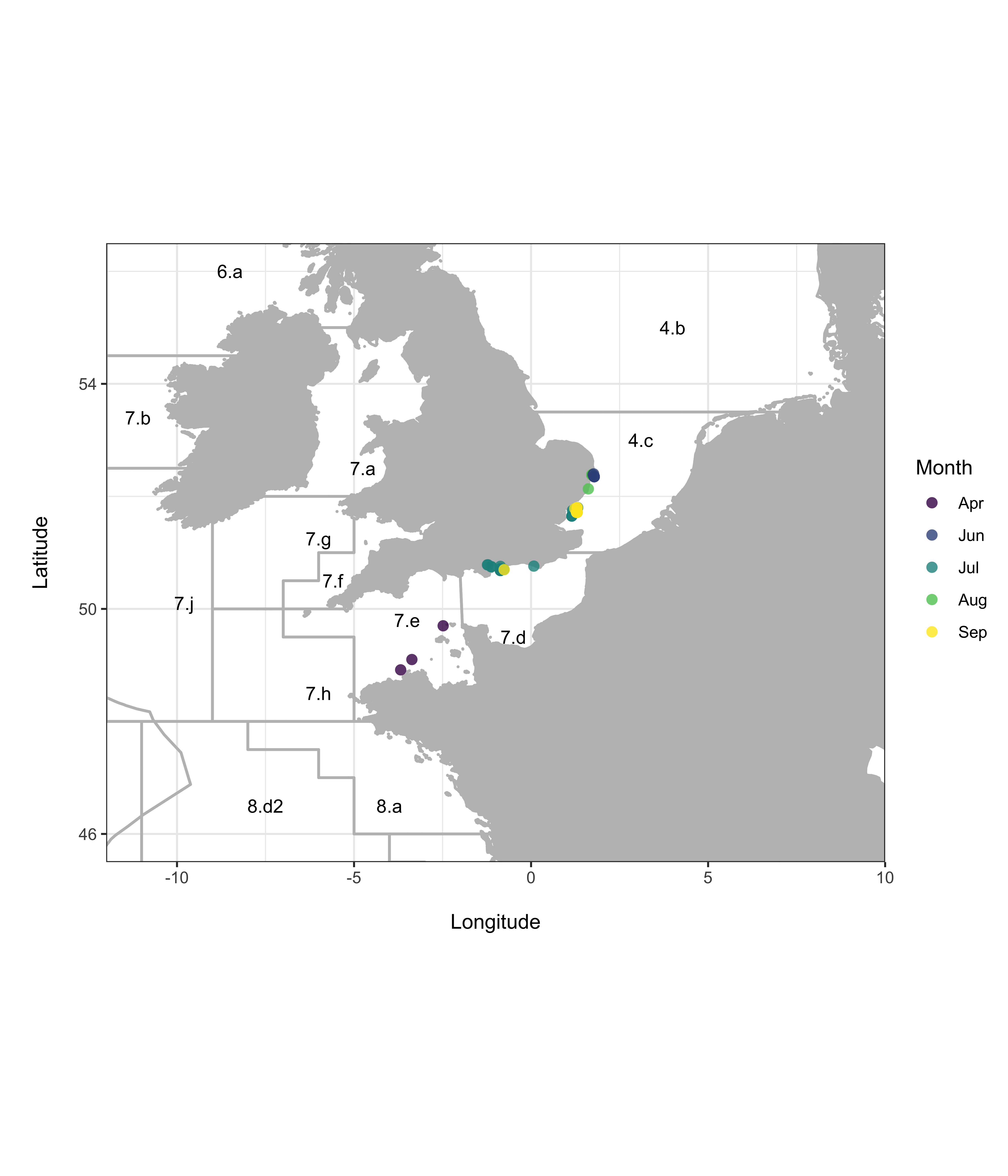

Supplement: S4 Fig — Release locations of individual M. asterias tagged with electronic tags (n = 125) between January 2017 and October 2019. Points are coloured by month of release. ICES Divisions are labelled and correspond to the following areas: central North Sea (4.b), southern North Sea (4.a), eastern English Channel (7.d), western English Channel (7.e), Celtic Sea (7.f-h and 7.j), Irish Sea (7.a), west of Scotland (6.a), west of Ireland (7.b) and northern Bay of Biscay (8.a and 8.d2). (PNG) [file pone.0239480.s010.png]

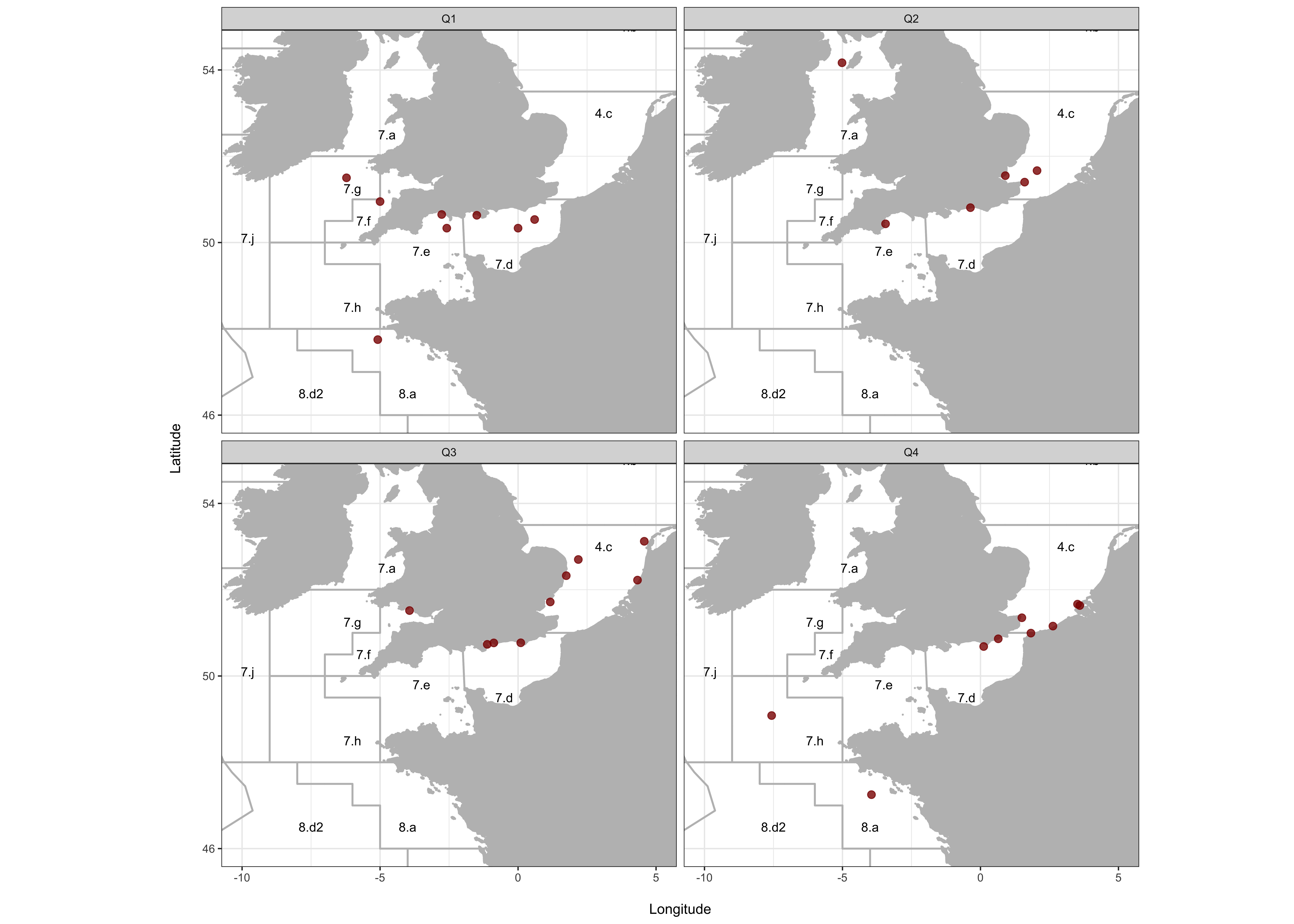

Supplement: S5 Fig — Recapture locations (red) of M. asterias by quarter (Q1, n = 8; Q2, n = 6; Q3, n = 10; Q4, n = 9), with ICES Divisions shown. (PNG) [file pone.0239480.s011.png]

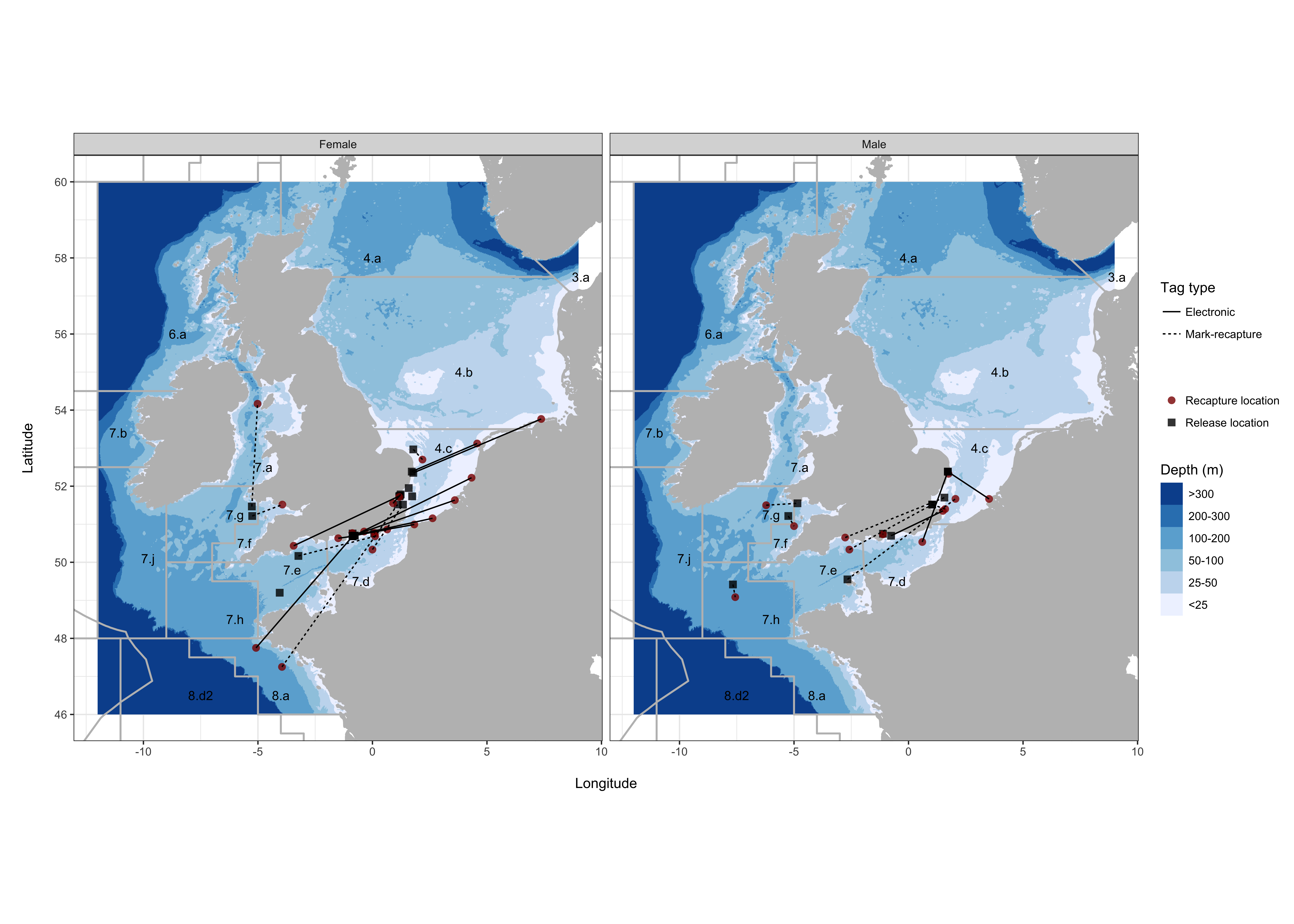

Supplement: S6 Fig — Release and recapture locations of M. asterias by tag type and sex (males, n = 14; females, n = 22), with ICES Divisions shown. (PNG) [file pone.0239480.s012.png]

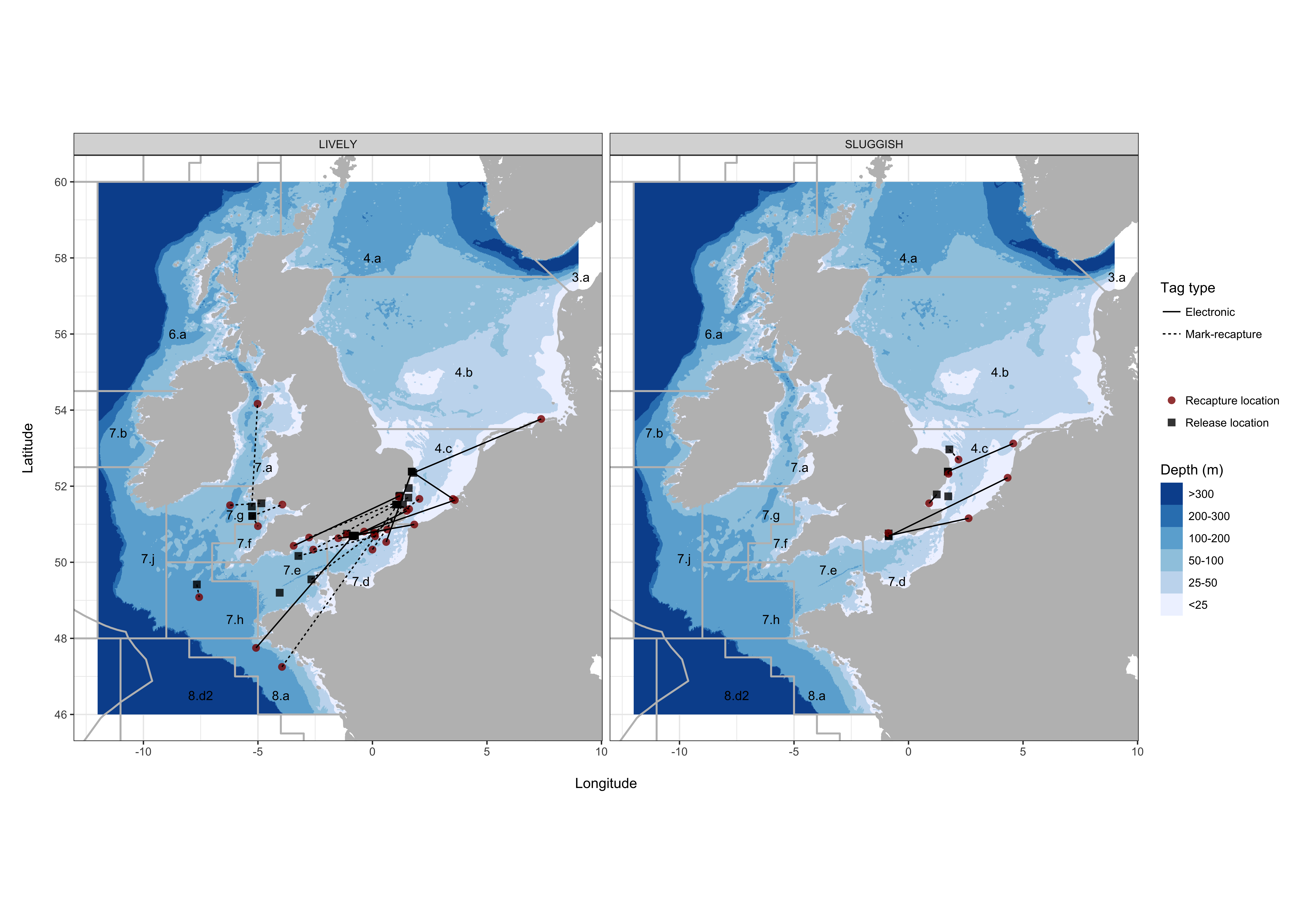

Supplement: S7 Fig — Release and recapture locations of M. asterias by tag type and condition at release (lively, n = 28; sluggish, n = 8), with ICES Divisions shown. (PNG) [file pone.0239480.s013.png]

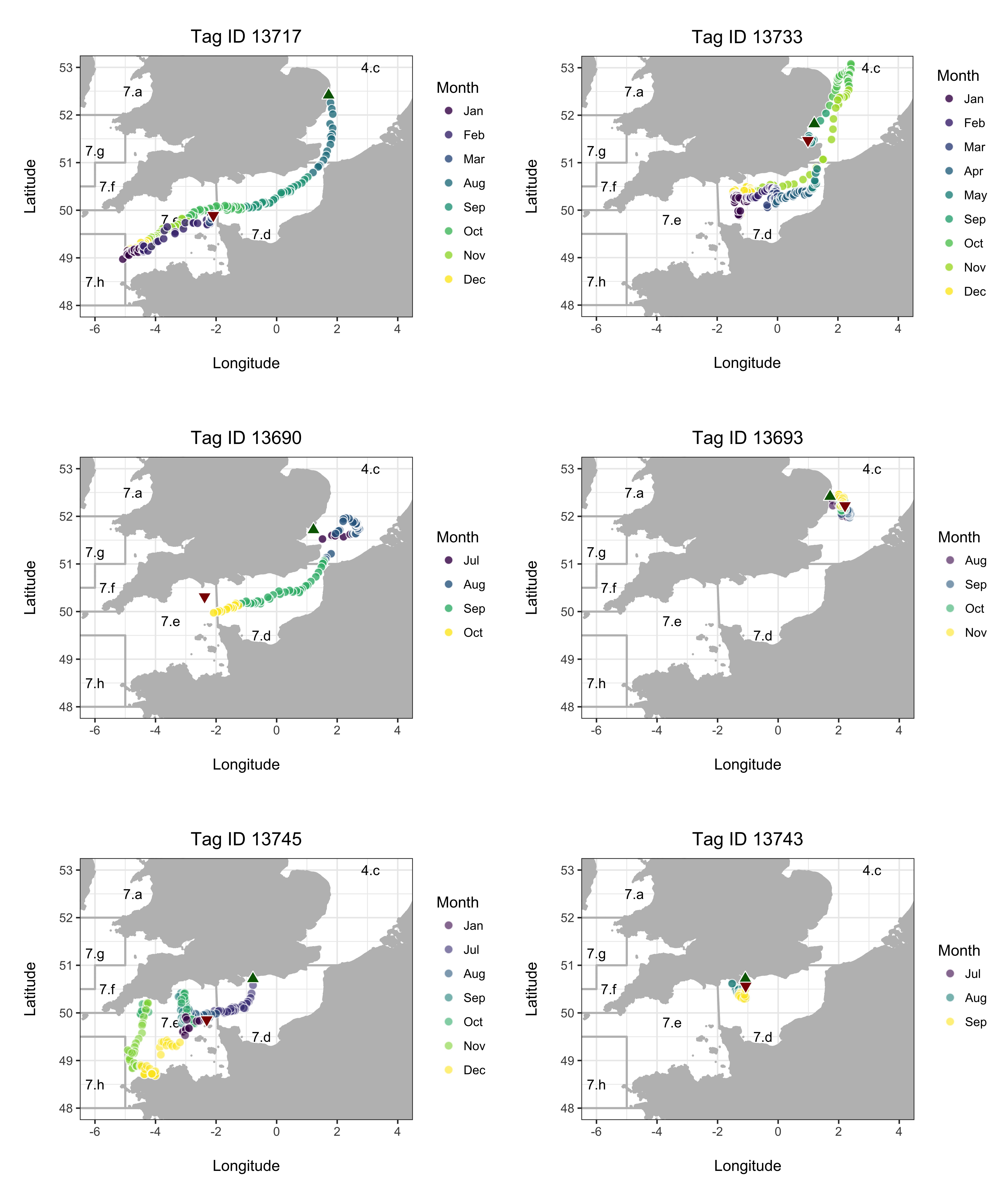

Supplement: S8 Fig — Movements of M. asterias tagged with electronic tags (n = 6). Locations in space and time are coloured by month to illustrate seasonality. No measurements were taken during June. ICES Divisions are labelled where appropriate. (PNG) [file pone.0239480.s014.png]
