# Supplementary material for: Horizontal and vertical movements of starry smooth-hound Mustelus asterias in the northeast Atlantic
Source: PLoS One. 2020 Oct 28;15(10):e0239480. doi: 10.1371/journal.pone.0239480 (PMC7592766; doi:10.1371/journal.pone.0239480)
Supplement: Attachment — Submitted filename: Response to reviewers.docx [file pone.0239480.s015.docx]

**Response to reviewer comments**

Please find below our response to reviewers for the paper entitled ‘Horizontal and vertical movements of starry smooth-hound *Mustelus asterias* in the Northeast Atlantic’. Reviewers comments appear first, and our responses follow within [RESPONSE: …]. As instructed, we have provided two versions of the MS, one which retains any tracked changes and a second unmarked version of the revised MS. Quoted line numbers refer to the MS with tracked changes included.

We were also asked to provide amended funding and competing interest statements, these can be found at the end of this document (see page 13). We declare that there have been no changes to our Data Availability statement i.e. all data will be made available (when accepted) via the Cefas Datahub. We have strived to ensure that the MS meets all of PLOS ONE’s style requirements including the inclusion of captions for Supporting Information files at the end of the MS document.

**********************************************************************************

*Academic Editor*

Thank you for submitting your paper, which has now been revised by two external reviewers. Both reviewers were supportive of the study and encouraged publication, one recommending major revisions and the second with just a very minor editorial revision.

I agree with both referees that this is a good study worth publishing in the scientific literature. As such, I am recommending major revisions. I would highly encourage you to prepare and submit a revised manuscript, taking into account all the comments and corrections provided by the reviewers. I would highlight in particular the main points raised by referee #1.

Thank you, and I am looking forward to receiving a revised version.

[RESPONSE: We thank the academic editor of PLOS ONE (Rui Coelho) and the external reviewers for their comments and handling of this paper. We agree that the reviewer’s comments were supportive and feel that the MS has been improved in light of their thoughtful and in-depth reviews. As highlighted, this is particularly true for reviewer #1. We feel that new MS is easier to follow (a main point highlight by reviewer #1) and more succinctly delivers our message of novel movement behaviour and stock structure in *M. asterias*.

We have also taken the opportunity to include an extra electronic time series (Tag 13693). This individual was included in the previous version of the MS (treated as a mark-recapture tag) but hadn’t been geolocated or described explicitly.]

**********************************************************************************

Reviewer #1

This manuscript uses various tagged methods to describe the horizontal and vertical movements of recently targeted shark species in the UK. The authors do a great job at setting up their study and providing rationale. However, in the methods and results, it becomes difficult to follow. I think this confusion can be fixed by trimming down a lot of the present text that describes the tagging and release information and summarizing all this information in a couple tables. The use of inconsistent terms throughout paragraphs in these two sections contributed to the confusion as well. I do think the low sample size is a slight issue, and although the authors mentioned that, I think too much emphasis has been put on individual behaviors and potential outliers instead of focusing on some of the similarities and trends shared among individual datasets. I also think comparing their results in the context of fishery independent and dependent data where sample sizes are much higher would improve this paper. With the changes suggested below, it definitely seems like the information from this study can complement the current knowledge on this species and also provide some new insight into various behaviors and stock structure.

[RESPONSE: We thank reviewer #1 for their comments and consideration, and greatly appreciate the effort made to refer to specific passages throughout. We feel that the in-depth review has helped improve the MS, in particular the methods and results sections. As detailed below, we have updated and corrected the manuscript accordingly.]

**Introduction:** The introduction is well written and clearly supports and provides the rationale for the objectives of this study. Below are a couple suggestions for this section.

**Comment L94**: I think the word large is a little extreme, may be better to remove it.

[RESPONSE: Removed.]

**Comment L104**: The way this sentence is written assumes the authors have already mentioned that knowledge of horizontal and vertical movements are lacking for this species; however, it is not mentioned anywhere in the introduction prior to this. The authors did provide why having that information is important though. Maybe mention vertical movement info is lacking in the same paragraph after that information is presented.

[RESPONSE: A good suggestion. We have added the following sentences on lines 104-106: “Despite this, very little is currently known about the vertical movements and depth utilisation of *M. asterias*. Electronic tagging of the species could therefore provide important insights into fundamental ecology and help inform both stock assessments and management.”]

**Comment L103:** I would argue that based on lines 82-86, horizontal information is not unknown, but that more detailed information is needed.

[RESPONSE: A good point. We have now amended the original text to address this on line 107. The text now reads “Given current unknowns surrounding the vertical movements of *M. asterias* and the uncertainties relating to horizontal movements, as well as the increasing abundance of this species in UK waters [19] and the increase in reported landings by UK vessels [18], the aims of this study were three-fold.”]

**Methods:** I think some reshuffling of this section would be beneficial. The number of individuals tagged with each tag type, in each region, and each time period should be moved to the results section. Leaving this information wouldn’t be that big of a deal if there weren’t so many values reported. There are multiple paragraphs dedicated to these values, which in my opinion would fall under the beginning of the results section. The detailed explanations of each tagging procedure could probably be shortened if space or word count are issues. The text is difficult to follow as is, especially if the reader is not looking at the pictures in the supplementary figures.

[RESPONSE: We agree – details of tag releases have now been moved to the start of the results section. The methods have been restructured to aid the flow of the text and provide the requested clarity. Furthermore, we have removed the tagging procedures relating to the attachment of the pDST and G6a+ tags as they were not utilised in the paper (along with their associated supplementary figures), thus substantially cutting down this ancillary text.]

**Comment L139**: Any reason why Condition is capitalized? Sex, maturity, and weight is not

[RESPONSE: It was meant to be the start of a new sentence – full stop added.]

**Comment L156-164**: It may be useful to cite studies that used each of these tag types just for reference

[RESPONSE: We agree. We have now added the following on line 146, ‘These DSTs have been deployed on a range of fish species in recent years (e.g. [37,38])’. Both papers deployed the Cefas G5 tag or the Cefas pop-off G5 tag on free-roaming marine fish.]

**Comment L222**: should be where not were

[RESPONSE: Corrected.]

**Comment L224-242**: There should probably be mention of whether the depth and temperature data were summarized or aggregated at all (e.g. by hour), unless data were summarized every 10 mins. If so, please explicitly state that. Also, were data from all three types of electronic tags used for this analysis? What is the error associated with these positions? This is something that should be stated especially since they are estimated positions.

[RESPONSE: The depth and temperature data were summarised every 10 minutes for all electronic tags which had useable data. We have amended the text on line 188 to state ‘Prior to geographical reconstruction, each fish’s depth and temperature measurements were visually assessed and down sampled to a 10-minute resolution’. We have also added ‘Only electronic tags recovered with sufficient data were considered for geographical reconstruction’ on line 192 to provide further clarity.

The question ‘were data from all three types of electronic tags used for this analysis?’ is now explicitly addressed on line 148: “The geographical and behavioural analyses presented here only consider data extracted from the G5 DST tags, so only these tags are described in detail. Returns from the other two electronic tags (pDSTs and G6a+) are presented as recapture positions only.”

The error associated with each daily geolocation varies by day, depending on whether there were daily tidal signals, or the number of temperature and depth matches to the underlying environmental data. Previously analyses on the mean error associated with this geolocation approach have found that it was approximately 8 km per day (Hobson et al. 2009), which is comparable to error from Argos locations (Hays et al. 2001b) and is an order of magnitude better than some light-based geolocation approaches (Teo et al. 2004). To provide some clarity on this matter, we have added the following sentences on line 184: “The error associated with each geographical position will vary on a daily basis depending on the certainty in the positional estimate, for example, whether or not a tidal signal was present in the depth data. For further details we refer the reader to [39] and [43].’]

**Commen**t **L256**: I know it may be obvious, but authors should be clear that for the vertical and seabed analyses they only used data from the electronic tags.

[RESPONSE: It is obvious, but we agree that some clarity might be needed. We have added the following on line 201: ‘Vertical and depth analyses only consider data from electronic tags.’]

**Comment L269**: I think this assumption can be dangerous, especially if this species makes a diel migration into shallower waters but remains on the bottom. If this isn’t the case I would state that.

[RESPONSE: We agree and feel that the reviewer has raised an interesting point. To provide clarity we have added the following text on line 221: “This approach assumes that individuals do not move into substantially shallower water within a 24-hour cycle.” We have also made a notable effort to amend the text within the discussion (see comments below).

**Comment L270**: Some may argue that Vertical Speed is also a Vertical movement behavior and thus be included in this section.

[RESPONSE: We agree but also feel that keeping them separate makes the text easier to follow.]

**Comment L271**: Authors should explain why they decided to go with a nonparametric approach.

[RESPONSE: We don’t think that this is a necessary step. Non-parametric approaches are commonly used to geolocate fish in coastal areas because parametric alternatives (e.g. Kalman filters) would assign non-zero probabilities to dry land and fish don’t move over dry land. This point would be appropriate if we were considering the movement of fish in the open ocean, but this isn’t the case here. Furthermore, the approach of Pedersen et al. (2008) has been widely used in UK and European waters to address the geolocation problem and we see no reason why further justification is needed.]

**Comment L274**: I think the authors mean reverse not reserve.

[RESPONSE: Text amended.]

**Results:** The Recaptures, Horizontal movement sections can be substantially reduced. They are also extremely hard to follow as I read from one paragraph to the other because the information being described is very similar among paragraphs. I think having one or two tables describing most of this information would suffice and the authors could simply highlight the most important trends. I don’t think it is necessary to discuss results of individuals (particularly for mark-recapture fish). The authors also partially describe some tagging locations in this text as well which is repetitive from the methods section. I think breaking down the number of recaptures by Quarter is fine but there are times when it is broken down by month which is inconsistent and feels too detailed. As I mentioned further up, it would be best to summarize the tagging and recapture trends in a couple tables and substantially reduce the text.

[RESPONSE: We agree and thank the reviewer for highlighting these points. As advised, we have substantially reduced the recaptures and horizontal movement sections, removing over 400 words. We have shifted a lot of the finer details in regard to release and recapture information to supplementary tables and tried our best to ensure that only the key trends are described. We have moved away from describing individual movements in nearly all instances and now only use them to highlight key take home messages. We have also taken this approach for the electronic tag descriptions (now labelled as geographical locations).

Overall, we feel these amendments have greatly increased the readability and flow of the result section.]

**Comment L281-284:** I think it would be best to weave the summary of fish tagged in this section. It would also probably end up being more concise as well.

[RESPONSE: We agree, the summary of fish tagged is now considered as part of the results section and has greatly helped the flow of the text. This section is titled ‘Releases’ can now be found on line 235.]

**Comment L291**: It might be helpful to define all the Quarters.

[RESPONSE: Definitions of each quarter have now been provided on lines 239 and 258-260.]

**Comment L292**: Should be where not were

[RESPONSE: Corrected.]

**Comment L293**: Putting this release information further emphasizes the need to put the other tagging numbers in this section. Also since the authors are comparing characteristics between release and recapture (e.g. dates, days at liberty), it might also be informative to include the average increase in length and weight.

[RESPONSE: We agree and feel the MS has benefited greatly from this suggestion. We also agree that an average increase in length and weight would be informative, however, we have chosen not to include it as the number of fish returned with this information was low (n = 9). Moreover, because it can often take time to physically recover a carcass, the length at recapture is often less than the length at release due to the freezing process. This severely limits the usefulness of this result.]

**Comment L297**: failure should be plural

[RESPONSE: Corrected.]

**Comment L301-305**: It is a little confusing here because you already talk about recaptures in different quarters on line L291.

[RESPONSE: A very valid point. In the new amended version of the results section this is no longer an issue. Recapture information by quarter is now only described on lines 257-260 within the ‘Recaptures’ section.]

**Comment L334**: There is no description of the tag and recapture symbol in the figure caption.

[RESPONSE: We have added ‘Release and recapture locations of *M. asterias* are plotted as triangles and inverted triangles, respectively’ to the figure caption.]

**Comment L338-362**: Descriptions on the individual level I think is not necessary. I think describing trends (directional movements, area use, and temporal movements) among these individuals would result in a much clearer section. In addition, the authors provided a figure (Figure 3) that group individuals based on these trends, so it appears to describe the results in the context of those trends would be better. Also there should be a clear description of what each time step position represents, particularly in the figure (every 10 mins as described in the methods? every hour? every day?).

[RESPONSE: We agree that descriptions at the individual level isn’t a necessary step and results in a lot of ancillary text. As advised, we now describe geographical movements in the context of trends and not individual-level details. The new text can be found on lines 279-300.

Each time step represents a day (24 hours) and although we have used the terms ‘daily’ and ‘each day at liberty’ in the geographic location section of the methods, we see the reviewers point and have added the following text to the figure legend on lines 290-295: “Each location represents an estimated geographical position per day (24 hours)”. We have also added the word ‘Daily’ on line 290.]

**Comment L364-372**: Similar to above, this much information on the individual level is not necessary. I think it is okay to show an individual example, but to get into this level of detail takes away from the important trends among individuals described further down. Also further up in the results you use Quarters to describe time and now you are using spring and winter and months instead. Make sure to change this and be consistent throughout.

[RESPONSE: We agree and have now removed the individual-level detail from the section. The text now simply describes the general seasonality shown in the example figure and leads nicely into the important trends among individuals in the next paragraph. The new text is detailed on lines 302-305.

We have also been through the whole the MS and now refer consistently to quarters as opposed to seasons.]

**Comment L379**: Again, I don’t think it is necessary to included individual metrics like the depth and the exact date for specific individuals. Does this level of information really help get at the three objectives mentioned at the end of the introduction? You are using seasons here, not Quarters, please change one or other for consistency.

[RESPONSE: We agree that in general a shift away from individual-level metrics has greatly increased the readability and flow of the results section. However, here we have to disagree. The finding that an individual *M. asterias* has moved into depths that exceed 100 m is a novel one and therefore this needs to be detailed. As does the thermal range in which the species resides. Consequently, we feel that these points help achieve objective two: novel insights into the depth utilisation and thermal habitat of the species.]

**Comment L395**: The fact an individual went this fast is cool, but doesn’t help you understand vertical behavior of this shark species as a whole. I understand you have a low sample size, but still think it is important to concentrate the vast majority of text in the results on trends on the species level.

[RESPONSE: We agree and have removed this text.]

**Comment L399**: Sounds awkward, please reword.

[RESPONSE: The original text has been amended and now reads “Average vertical speeds were much higher during the night (0.27 m min^-1^) than during the day (0.13 m min^-1^; Student’s t-test: t = 6.4, df = 47.4, p-value < 0.001).”]

**Comment L391-406**: Multiple times authors used some sort of t-test here to compare day and night, but there is no description of this test in the methods section. Is it non-parametric? If not, why is it parametric, while a nonparametric approach was used for geographic location?

[RESPONSE: The t-test is a simple Student’s t-test and has been used to compare depth, vertical speed and proximity to the seabed during the day and during the night. We agree that this needs to be stated in the methods section and a new subsection titled statistical analysis has been added on line 231.

We see no reason why there should be synchrony in terms of the statistical nature of the geolocation approach and a simple test to assess whether fish were significant deeper during the day or night. If the reviewer has an explicit reason for this, we are happy to discuss further, however, at the moment this seems unnecessary.]

**Comment L414**: There is no acronym for reverse diel vertical migration in figure caption.

[RESPONSE: Added.]

Discussion: The discussion was written fairly well. I think the fact that the results were difficult to follow made it a little difficult to follow at least at the beginning. I think it also would improve the discussion if the authors expanded a little more on some of their ideas.

**Comment L421**: I think this statement is obvious and doesn’t need to be stated here.

[RESPONSE: We agree and have adjusted the MS accordingly.]

**Comment L450**: Where are female skates known to move further distances? Do all skates species do this? **Comment L450-451**: Any speculation or hypothesis why females travel further than males?

[RESPONSE: The paper we cited was from the British Isles, which is now indicated to provide further clarity. There are currently insufficient studies on skate tagging to say whether this type of sex-biased dispersal in common in skate, but it has been noted for *R. binoculata* (King & McFarland 2010). We have now added this reference to the text. In terms of reasons why this may happen, it could related to body size (given that female elasmobranchs often attain a larger size than males) and/or reproductive requirements, with females seeking to utilise optimal environments (in terms of temperature and prey availability) to maximise ovarian and embryonic development, whilst also having moving to other habitats for giving birth. To address all these points, we have modified the original text on lines 371-379 to: “Whilst our findings are based on a limited sample size (n = 36), it is noteworthy that recent studies on skates have found that females undertake much longer-distance movements in waters surrounding the British Isles [36] and in the northern Pacific [57]. Similarly, mark-recapture studies by Francis [58] revealed that female rig *Mustelus lenticulatus* around New Zealand travelled further than their male conspecifics. The reasons why females may disperse over larger distances than males could relate to body size (given that female elasmobranchs often attain a larger size than males [19]) and/or reproductive requirements, with females seeking to utilise optimal environments (in terms of temperature and prey availability) to maximise ovarian and embryonic development [59].”]

**Comment L473-478**: This is a little confusing because at first you mention individuals that migrate, but then you focus on one particular individual. I think this would be clearer if these trends were articulated better in the results.

[RESPONSE: Thanks for highlighting this. The one particular individual provides direct evidence of philopatric behaviour and therefore merits inclusion. We hope that the new results section aids clarity of this matter. We have also made several small amendments to this section to more appropriately consider the extra electronically tagged fish.]

**Comment L506**: I think distributions from this study should be more directly compared to what the landings data suggest. Expanding on this may help.

[RESPONSE: We agree that comparative studies of these movements in relation to landings data would be beneficial, however, this is beyond the scope of the current paper. Studies that consider landings data have been supplied in the text (e.g. McCully *et al.* (2015)) and we have now expanded upon the them to provide greater detail. The following text can now be found on lines 426-435: “However, release dates in Q3 (S5 Table), landings records [18,19,63] and past modelling work [64] suggest otherwise, highlighting that *M. asterias* are abundant in these areas year-round. Furthermore, recent work by McCully Phillips and colleagues [63] demonstrates that European landings of *M. asterias* in 2018 were primarily made from the eastern (33%) and western English Channel (29%), and UK landings (2014-2018) were made throughout the year in these areas. Such trends illustrate that migration into the deeper waters of the western English Channel and Celtic Sea might not be the only behavioural strategy in *M. asterias*, with some individuals opting to overwinter in eastern English Channel and southern North Sea. Given our limited knowledge of the species, residency in the English Channel may be a long-standing characteristic of *M. asterias* ecology.”]

**Comment L516**: It is important to cite some studies that found this trend in species for your study area here.

[RESPONSE: We agree. We have now updated the references to reflect changes that have been observed in the North Sea as well as the south-west. The text now reads: “For example, several studies have now linked rising sea temperatures and other environmental covariates to both a northward displacement and a deepening of commercially important fish stocks in the North Sea [65–67], as well as northward shifts in some fish distributions to the south-west of the British Isles [68,69]”]

**Comment L518-522**: This is all summarized results. I don’t have a problem with some repetition between the results and discussion but this is almost a whole paragraph of repeated results.

[RESPONSE: Thank you for highlighting this, we have removed some of the repetition from this section. The text now reads: “During Q3 *M. asterias* were found to occupy warm shallower waters, whereas during Q4 and Q1 their depth utilisation is markedly deeper and experienced temperatures are reduced.”]

**Comment L531**: remove in spatial distribution

[RESPONSE: Text amended.]

**Comment L531**: I think providing some temperature reference between the southern North Sea and western English Channel would help the reader understand the thermal differences between the two areas. As it stands, latitudinally they don’t appear too different.

[RESPONSE: The reviewer is correct in noting that some international readers may not appreciate the pronounced temperature differences between the southern North Sea and English Channel. References have been added to this sentence, which highlight the temperature difference in the English Channel compared to the North Sea. The text has been amended to: “Sea surface temperature in the southern North Sea peaks in August [70], and consequently individuals may be residing in these areas until the water temperature reaches some critical threshold which triggers a southward shift. This seasonal shift in location during the winter and spring would then presumably allow individuals to exploit the deeper and warmer waters of the western English Channel [71,72].”]

**Comment L547-551**: Split into multiple sentences.

[RESPONSE: Text amended.]

**Comment L550**: What if they aren’t moving off the bottom but moving to shallower waters to feed on crustaceans, but it looks like they are moving up in the water column?

**Comment L562**: Again, unless the sharks aren’t moving off the bottom, but just moving to shallower habitats, the trawl would still catch them unless the habitat is too shallow to fish. Is there not trawl data on M. asterias catch at night? I wonder if depth of catch of M. asterias differs between day and night?

[RESPONSE: These comments raise a very interesting point. Indeed, our method for calculating the proximity to the seabed does not account for the possibility that fish may move into shallower waters within a 24-hour cycle. To acknowledge this, and its possible effects to our conclusions (re. vertical behaviour and catchability) we have made several amendments to the text which can be found on lines 462-490.]

**Comment L573-575**: Would be nice to include something about what type of work would help address this question…acoustic telemetry? Genetic study?

[RESPONSE: This is an important point and the text on lines 498-501 has been amended to read: “Despite this, it remains uncertain whether the two sub-populations mix, and future work should prioritise more targeted tagging programmes (e.g. tagging of *M. asterias* in the Bristol Channel and Irish Sea during Q2 and Q3 and in the Celtic Sea in Q4), as well as studies of genetic structure [82], vertebral microchemistry [83] and isotopic signatures [84].”]

**Comment L587**: Back to Quarters again, instead of seasons.

[RESPONSE: Quarters are now used throughout, albeit some reference to seasonality is made.]

**Figures**: I imagine that if accepted, figures will be uploaded at a higher resolution because right now, it is difficult to read the letters on the figures on the pdf document.

[RESPONSE: Yes, all figures will be uploaded as separate .tiff files with adequate resolution (300-600 dpi). We agree that the conversion to pdf during the review process has made the figures hazy and a little difficult to read. If this continues to be a problem, we are happy to increase the text size on each figure.]

**Comment Figure 1.** It would be helpful to include the geographic names on the map for readers not from the UK (e.g. Celtic Sea, southern North Sea, etc)

[RESPONSE: A very useful suggestion and we agree that the inclusion of more information may help readers not from the UK. That said, we feel that the addition of geographic names to the map object will result in an overly cluttered plot. ICES Divisions are already clearly labelled, and these correspond explicitly to geographical areas. They also provide a necessary link to stock assessment and management. Consequently, we have added the following text to the figure legend: ‘ICES Divisions are labelled and correspond to the following areas: northern North Sea (4.a), central North Sea (4.b), Skagerrak (3.a), southern North Sea (4.c), eastern English Channel (7.d), western English Channel (7.e), Celtic Sea (7.f-h and 7.j), Irish Sea (7.a), west of Scotland (6.a), west of Ireland (7.b) and northern Bay of Biscay (8.a and 8.d2)’. We hope that this helps address the reviewer’s comment.]

**Comment Figure 4.** Why not combine the depth and temperature figure into one plot? That is commonly done in many archival studies.

[RESPONSE: A good point – Figure 4 has now been adapted and depth is plotted in a colour gradient of temperature (from red to blue).]

**Comment Figure 7**. I assume eVM in the figure legend should be nVM.

[RESPONSE: Yes, thanks for spotting this. We’ve corrected the error.]

**Comment Supplementary Data**: Supplementary Table 2 may be better to just include May as its own row and put 0 for Number of fish tagged.

[RESPONSE: Row for May added.]

**********************************************************************************

Reviewer #2

"Horizontal and vertical movements of starry-smooth hound *Mustelus asterias* in the Northeast Atlantic" is a novel study that provides valuable information on a data-limited species. Information on ecology and species behaviour is essential in fisheries management, therefore this manuscript is a good contribution to fill knowledge gaps on these issues for the starry smooth-hound. The manuscript meets the requirements for publication; results are presented in a straightforward and organized way and it is well written.

[RESPONSE: We thanks reviewer #2 for the review and comments.]

**Comment**: Lines 321, 328, 336: replace "spilt" with "split"

[RESPONSE: Text amended.]

*********************************************************************************

Amended Funding Statement:

This work was funded by the European Maritime and Fisheries Fund project ENG 1395, Defra and Cefas’ science development scheme. The funders provided support for consumables, field work and staff time for all authors (CAG, SRW, JFS, JRE, DAR and SMP), but did not have any additional role in the study design, data collection and analysis, decision to publish, or preparation of the manuscript. The specific roles of these authors are articulated in the ‘author contributions’ section.

Amended Competing Interests Statement:

Several of the authors are/were employed by Cefas, however, we confirm that this does not alter our adherence to PLOS ONE policies on sharing data and materials.
